# The Directionality of Fronto-Posterior Brain Connectivity Is Associated with the Degree of Individual Autistic Traits

**DOI:** 10.3390/brainsci11111443

**Published:** 2021-10-29

**Authors:** Luca Tarasi, Elisa Magosso, Giulia Ricci, Mauro Ursino, Vincenzo Romei

**Affiliations:** 1 Centro Studi e Ricerche in Neuroscienze Cognitive, Dipartimento di Psicologia, Alma Mater Studiorum—Università di Bologna, Campus di Cesena, 47521 Cesena, Italy; luca.tarasi2@unibo.it; 2 Department of Electrical, Electronic, and Information Engineering “Guglielmo Marconi”, University of Bologna, 47521 Cesena, Italy; elisa.magosso@unibo.it (E.M.); giulia.ricci29@unibo.it (G.R.); mauro.ursino@unibo.it (M.U.); 3 IRCCS Fondazione Santa Lucia, 00179 Rome, Italy

**Keywords:** autism spectrum disorder, autistic quotient, EEG, Granger causality, bottom-up and top-down connectivity

## Abstract

Altered patterns of brain connectivity have been found in autism spectrum disorder (ASD) and associated with specific symptoms and behavioral features. Growing evidence suggests that the autistic peculiarities are not confined to the clinical population but extend along a continuum between healthy and maladaptive conditions. The aim of this study was to investigate whether a differentiated connectivity pattern could also be tracked along the continuum of autistic traits in a non-clinical population. A Granger causality analysis conducted on a resting-state EEG recording showed that connectivity along the posterior-frontal gradient is sensitive to the magnitude of individual autistic traits and mostly conveyed through fast oscillatory activity. Specifically, participants with higher autistic traits were characterized by a prevalence of ascending connections starting from posterior regions ramping the cortical hierarchy. These findings point to the presence of a tendency within the neural mapping of individuals with higher autistic features in conveying proportionally more bottom-up information. This pattern of findings mimics those found in clinical forms of autism, supporting the idea of a neurobiological continuum between autistic traits and ASD.

## 1. Introduction

Autism spectrum disorder (ASD) is a biologically-based condition characterized by the presence of restricted and rigid behavior (fixations on certain activities, or specific routines or rituals) and deficits in socio-communication abilities (inappropriate social interaction, deficiencies in developing and maintaining social relationships) [1]. The whole conceptualization of this condition has changed in recent years: the dichotomous (i.e., absent or present) approach has been replaced by a dimensional (continuum) perspective [2]. This change in perspective stems from the presence of a constellation of heterogeneous clinical manifestations that can be placed in the autistic boundaries [3]. In recent years, the dimensional conceptualization of autism has been further extended. Indeed, several lines of research claim that there are no definite boundaries separating autism from the rest of the general population, as symptoms span from overt clinical manifestations to trait-like expression of this condition (autistic traits) [4]. The existence of an extended autistic phenotype is confirmed by studies showing a similar genetic basis in individuals within the general population who express autistic traits and in those with diagnosed autism [5]. Furthermore, individual differences in autistic traits in the general population show substantial heritability, which is consistent with genetic evidence suggesting the heritability of autism [6]. Indeed, relatives of children with ASD exhibit a subclinical set of autistic-like features in social and communication abilities to a greater extent than relatives of neurotypical children. These subclinical differences observed in people genetically close to ASD subjects are generally considered to constitute the broader autism phenotype (BAP) [7]. In addition to genetic commonalities, similarities between ASD and individuals with high autistic traits are also evinced at the neurocognitive level. Similar to clinical ASD individuals [8], individuals with a high level of autistic traits are faster and more accurate in tasks measuring visuospatial ability, such as the Embedded Figures Test and the Block Design Task [9], and both show an enhanced reliance on details in perceptual processing [10] as well as a preference for predictability [11]. Neuroscientific research has been studying the neural determinants of autism for decades. Individuals within the general population with high autistic traits represent an opportunity to increase knowledge about the neurobiological mechanisms underlying autism, limiting confounding factors such as medication, chronicity, and comorbidities that are present in overt clinical forms.

A large body of evidence has shown a close link between brain connectivity anomalies and autistic spectrum symptoms. Studies using functional magnetic resonance imaging (fMRI) have consistently indicated widespread impairment of white matter projections in ASD, mainly in the corpus callosum, bilateral frontal–occipital fasciculus, right arcuate fasciculus, and right uncinate fasciculus [12]. Similarly, studies investigating connectivity patterns on faster time scales using magnetoencephalography (MEG) and electroencephalography (EEG) assert that widespread connectivity alterations are distinctive biomarkers of ASD [13,14]. A study using a large cohort of children (430 ASD and 554 controls) showed that EEG coherence analysis between brain areas can identify children with ASD with a classification success of 86% [15]. However, the nature of these connectivity anomalies is still under debate. Several studies in the clinical ASD population have shown a weakness in functional connectivity between brain areas during cognitive tasks [16,17], leading to the development of the *underconnectivity theory* of autism [18]. In a review analysis, 26 out of 33 neuroimaging studies analyzed showed the presence of reduced or lack of connectivity in ASD, especially in long-range connections [19]. However, there is a whole branch of literature that points in a different direction showing the prevalence of strong brain hyper-connectivity in individuals within the autistic spectrum. Supekar et al. [20] found functional hyper-connectivity between both proximal and distant anatomical regions, suggesting the presence of both short- and long-range aberrant integration in children with ASD. Moreover, abnormal brain connectivity could also predict autistic symptoms, as children characterized by the presence of brain hyper-connectivity were more impaired in social domains and manifested greater autistic symptom severity [21].

These apparently contradictory results could be disambiguated by taking into account the directionality of the considered connections. Specifically, functional connectivity in ASD might be abnormally increased when mediated by the feed-forward pathways, but abnormally decreased when mediated by the feedback pathways. This pattern of over-representation of feed-forward information flow and weak top-down signaling would be congruent with the sensory peculiarities observable in ASD individuals. In fact, compared to control participants with typical development, children with ASD are more influenced by bottom-up visual information (conveyed through feed-forward pathways) [22] and less constrained by top-down prior information (conveyed through feedback pathways), leading to an increased veridical representation of the external world [23]. Indeed, Takesaki et al. [24] showed that increased occipito-frontal connectivity in the frequency band involved in the spreading of feed-forward information (i.e., gamma band) was associated with higher performance in a visual task in ASD participants. Additional strong support for this proposal comes from an MEG study conducted by Khan et al. [25]. The authors showed that during somatosensory stimulation, ASD participants were characterized by the presence of increased long-range feed-forward connectivity from the primary (S1) to secondary (S2) somatosensory cortex at 25 Hz (i.e., beta band), and this strengthened connectivity was correlated with multisensory processing abnormalities. Complementarily, the ASD participants were characterized by reduced long-range feedback connectivity [26]. The weakness in top-down signaling in ASD was confirmed by Seymour et al. [27] using a visual task: V4-to-V1 feedback connectivity in the alpha band was reduced in individuals on the autism spectrum compared to healthy controls and the strength of this feedback connectivity was inversely related to the autistic quotient score. Moreover, ASD individuals showed a systematic reduction in posterior alpha amplitude and enhanced frontal theta activity relative to their neurotypical control group [28,29].

However, although autistic traits are distributed to varying degrees in the general population, it remains unclear whether the peculiarities in brain organization found in ASD patients are also present to a lesser degree in non-clinical individuals with high autistic traits. The first studies conducted analyzing this subclinical population showed that higher levels of autism features are related to decreased white matter integrity in tracts associated with visual processing and increased cortical volume in areas related to working memory [30]. Other studies showed that having higher autistic traits was correlated with white matter alteration in the inferior fronto-occipital fasciculus [31] and a negative correlation was found between individual autistic traits and the efficiency of brain networks in an EEG study [32]. However, to date, we are not aware of other studies that have investigated the presence of a direction-dependent pattern of cortical connectivity (matching that observed in ASD) also in the general population with elevated autistic traits.

To fill this gap in the literature, in the present resting-state EEG study we assessed the presence of a differentiated connectivity pattern according to the individual autistic trait, using directional connectivity indices (e.g., Granger analysis). This analysis estimates the causal relationship between distinct brain area activity, measuring the extent to which one time series can predict another [33,34] and if so in which frequency band [35]. Via this data-analysis technique, we sought to determine whether the imbalances between top-down and bottom-up contributions observed in clinical forms of autism would reverberate in the general population according to the individual autistic trait.

## 2. Material and Methods

### 2.1. Participants

Twenty participants (14 female; age range 21–30) took part in the study. All participants signed a written informed consent prior to taking part in the study, which was conducted in accordance with the Declaration of Helsinki, and approved by the Bioethics Committee of the University of Bologna. All participants had no neurocognitive or psychiatric disorders. All participants completed the Autism-Spectrum Quotient test (AQ) [4]. The AQ is a self-report consisting of 50 items widely used to measure autistic traits in the general population. The AQ is divided into 5 subscales. Each subscale addresses a psychological domain implicated in ASD: communication, imagination, social skills, attention to detail, and attention switching. The sum of the scores obtained in each subscale provides a global score, with higher values indicating higher levels of autistic traits. We used the original scoring methods, converting each item into a dichotomous response (agree/disagree) and assigned the response a binary code (0/1). With this scoring procedure, the maximum score obtainable is 50. A cut-off score of 32 correctly identifies 76% of patients with ASD [36], whereas in the general population the average score is around 17 [37]. In the present study, the total score of the AQ was considered and the Italian version of the AQ was adopted [38].

### 2.2. EEG Acquisition and Preprocessing

Participants comfortably sat in a room with dimmed lights. EEG was recorded at rest for two minutes, while participants kept their eyes closed. A set of 64 electrodes was mounted according to the international 10–20 system. EEG was measured with respect to a vertex reference (Cz) and all impedances were kept below 10 kΩ. EEG signals were acquired at a rate of 1000 Hz. EEG was processed offline with custom MATLAB scripts (version R2020b) and with the EEGLAB toolbox [39]. The EEG recording was filtered offline in the 0.5–70 Hz band. The signals were visually inspected, and noisy channels were spherically interpolated. The recording was then re-referenced to the average of all electrodes. Subsequently, we applied the independent component analysis (ICA), an effective method largely employed for removal of EEG artefacts. Components containing artifacts that could be clearly distinguished from brain-driven EEG signals were subtracted from the data.

### 2.3. Cortical Source Reconstruction and ROI Definition

Since we were interested in connectivity analysis, cortical source activity was reconstructed starting from pre-processed EEG signals. To this aim, intracortical current densities were estimated using the Matlab toolbox Brainstorm [40]. Firstly, to solve the forward problem, a template head model (ICBM 152 MNI template) was used as implemented in OpenMEEG software [41] via the boundary element method. This provides three layers representative of the scalp, the outer skull surface, and the inner skull surface, and realistic anatomical information.

Then, the sLORETA (standardized Low Resolution Electromagnetic Tomography) algorithm was used for cortical source estimation. sLORETA is a functional imaging technique belonging to the family of linear inverse solutions for 3D EEG source distribution modeling [42]. Specifically, this method computes a weighted minimum norm solution, where localization inference is based on standardized values of the current density estimates. This method provides an instantaneous, distributed, discrete, linear solution with the property of zero dipole-localization error under ideal (noise free) conditions. For sources estimation, the choice of constrained dipole orientations was made, which models each dipole as oriented perpendicularly to the cortical surface.

Hence, for each participant, we reconstructed the resting-state time series of current densities at all cortical voxels (15,002 voxels).

Then, the cortical voxels were grouped into cortical regions according to the Desikan–Killiany atlas [43] provided in Brainstorm, which defines 68 regions of interest (ROIs) as reported in Table 1. At each time point, the mean activity of the voxels belonging to a particular ROI was used as a waveform of the cortical activity in that ROI. It is worth noting that some possible inaccuracies in voxel-level source reconstruction deriving from the use of a template head model for all participants (instead of subject-specific head model) were mitigated by considering the average behavior at ROI level.

### 2.4. Granger Causality Analysis

*Time-Domain Granger Causality*—Once the time waveform in each cortical ROI was estimated (as described above), for each participant k (k=1,…,20) we evaluated the functional connectivity among the ROIs in order to investigate the relationship between the resting-state cortical communication and the AQ score. To this aim, we adopted the Granger causality (GC), which provides directional metrics of functional connectivity and is based on the autoregressive (AR) modeling framework. Let us indicate with $x_{k,i}\left[n\right]$ and $x_{k,j}\left[n\right]$ two time series representing the activity of two distinct cortical ROIs ($ROI_{i}$ and $ROI_{j}$) for participant k. The Granger causality quantifies the causal interaction from $ROI_{i}$ to $ROI_{j}$ as the improvement in predictability of $x_{k,j}\left[n\right]$ when using a bivariate AR representation (based on past values of $x_{k,j}$ and also on past values of $x_{k,i}$) compared to a univariate AR representation (based only on past values of $x_{k,j}$). Mathematically, the following two equations hold for the univariate and bivariate AR model, respectively:

(1) $$x_{k,j}\left[n\right]=\overset{p}{\underset{m=1}{\sum }}a_{k,j}\left[m\right]x_{k,j}\left[n-m\right]+\eta_{k,j}\left[n\right]$$

(2) $$x_{k,j}\left[n\right]=\overset{p}{\underset{m=1}{\sum }}b_{k,j}\left[m\right]x_{k,j}\left[n-m\right]+\overset{p}{\underset{m=1}{\sum }}c_{k,ji}\left[m\right]x_{k,i}\left[n-m\right]+\varepsilon_{k,j}\left[n\right]$$

where index m represents the time lag; p is the model order; a, b, and c are the model’s coefficients (dependent on time lag); and the time series $\eta_{k,j}\left[n\right]$ and $\varepsilon_{k,j}\left[n\right]$ represent the prediction error of the AR model in each case. The variance of the prediction error quantifies the prediction capability of the model based on past samples: the lower the variance the better the model’s prediction. The GC from $x_{k,i}$ to $x_{k,j}$ is defined as the logarithm of the ratio between the variances of the two prediction errors, i.e.,

(3) $$GC_{k,ROI_{i}\to ROI_{j}}=\ln\frac{var\left\{\eta_{k,j}\left[n\right]\right\}}{var\left\{\varepsilon_{k,j}\left[n\right]\right\}}$$

The measure in Equation (3) is always positive, and the larger its value (i.e., the larger the improvement in the $x_{k,j}\left[n\right]$ prediction when using information from the past of $x_{k,i}$ together with the past of $x_{k,j}$) the larger the causal influence from $ROI_{i}$ to $ROI_{j}$. Similarly, the Granger causality from $x_{k,j}$ to $x_{k,i}$, $GC_{k,ROI_{j}\to ROI_{i}}$, is computed via the same procedure, building the AR models for the time series $x_{k,i}$.

For each participant k, we computed the two directed measures of GC for each pair of ROIs, overall obtaining 68 × 67 connectivity values. In all cases, the order p of the AR models was set equal to 30; this value was determined on the basis of a preliminary analysis where we tested different values for the order of the model, obtaining that the GC results did not change substantially for p≥30. Moreover, we tested the statistical significance of any single connection by evaluating whether the coefficients $c_{k,ji}$ in Equation (2) were jointly significantly different from 0 using the F-test (the routine for the GC F-test as implemented in Brainstorm was used). Only significant connections with a *p* value < 0.05 (Bonferroni corrected) were maintained in the subsequent analysis (all other connections were set to 0).

Then, for each participant, the total connectivity $\left(GC_{k}^{TOT}\right)$ was computed as the sum of all connection values, and each connection value normalized to the total connectivity, according to the following equations:

(4) $$GC_{k}^{TOT}=\underset{i}{\sum }\underset{j,j\neq i}{\sum }GC_{k,ROI_{i}\to ROI_{j}}$$

(5) $${\tilde{GC}}_{k,ROI_{i}\to ROI_{j}}=\frac{GC_{k,ROI_{i}\to ROI_{j}}}{GC_{k}^{TOT}}\cdot 100;\;\;\;\;\;\;\;\;\forall \;i,\;j\;\mathrm{with}\;i\neq j$$

In Equation (4), the summations extend over all the 68 ROIs; moreover, we have $\underset{i}{\sum }\underset{j,j\neq i}{\sum }{\tilde{GC}}_{k,ROI_{i}\to ROI_{j}}=100$ (i.e., from the adopted normalization, the sum of all connections among the 68 ROIs resulted to be equal to 100 within each subject).

Subsequently, for each participant k, we characterized each i−th ROI (i=1,…,68) in terms of the overall (normalized) connectivity outflowing from that ROI (${\tilde{GC}}_{k,ROI_{i}}^{OUT}$) and inflowing into that ROI (${\tilde{GC}}_{k,ROI_{i}}^{IN}$). Specifically, we computed

(6) $${\tilde{GC}}_{k,ROI_{i}}^{OUT}=\underset{j,j\neq i}{\sum }{\tilde{GC}}_{k,ROI_{i}\to ROI_{j}}=\left(\underset{j,j\neq i}{\sum }GC_{k,ROI_{i}\to ROI_{j}}/GC_{k}^{TOT}\right)\cdot 100$$

(7) $${\tilde{GC}}_{k,ROI_{i}}^{IN}=\underset{j,j\neq i}{\sum }{\tilde{GC}}_{k,ROI_{j}\to ROI_{i}}=\left(\underset{j,j\neq i}{\sum }GC_{k,ROI_{j}\to ROI_{i}}/GC_{k}^{TOT}\right)\cdot 100$$

In Equations (6) and (7), the summations extend over all the 68 ROIs. Based on the previous equations, ${\tilde{GC}}_{k,ROI_{i}}^{OUT}$ (named “output sum” for $ROI_{i}$) quantifies the contribution of the overall outflow from $ROI_{i}$ to the total cortical connectivity, and thus characterizes $ROI_{i}$ as a source of causal influence; ${\tilde{GC}}_{k,ROI_{i}}^{IN}$ (named “input sum” for $ROI_{i}$) quantifies the contribution of the overall inflow into $ROI_{i}$ to the total cortical connectivity, and thus characterizes $ROI_{i}$ as a sink of causal influence.

Then, in order to identify possible ROIs whose role as source or sink of causal inference was modulated by the AQ score, participants were divided into two classes, comprising participants with an AQ score below and above a given cutoff ($Class1=\left\{k:AQ_{k}\leq AQ_{th}\right\}$ and $Class2=\left\{k:AQ_{k}>AQ_{th}\right\}$, respectively), with the cutoff $AQ_{th}$ set to 17, since this value corresponds to the average AQ score in control subjects, according to the literature [37]. Next, the following steps were performed to determine the main source ROIs:

(i) For each $ROI_{i}$, we computed the statistical difference between the quantities ${\tilde{GC}}_{k,ROI_{i}}^{OUT}$ (k=1,…,20) in the two classes using the unpaired t-test and focused attention only on those with a *p* value < 0.05;

(ii) For each $ROI_{i}$, we computed the Pearson’s correlation coefficient between the score values $AQ_{k}$ and ${\tilde{GC}}_{k,ROI_{i}}^{OUT}$ (k=1,…,20). Only ROIs for which this analysis provided a correlation coefficient with an absolute value above 0.4, and for which the *p* value for testing the significance of the coefficient was less than 0.05 (computed using the t-distribution under the null hypothesis of no correlation), were taken into consideration.

Finally, for the following analysis step we focused only on the ROIs that simultaneously satisfied both criteria (i) and (ii) (hereafter defined as *AQ-modulated source ROIs*).

The same analysis (steps (i) and (ii)) was then repeated between the $AQ_{k}$ score values and ${\tilde{GC}}_{k,ROI_{i}}^{IN}$ (k=1,…,20), to define a set of *AQ-modulated sink ROIs*.

*Frequency-Domain (Spectral) Granger Causality*—For each participant k, k=1,…,20, we also computed the spectral Granger causality between each pair of ROIs, $ROI_{j}$ and $ROI_{i}$. Granger causality can be formalized in the spectral domain [35,44] starting from the joint bivariate autoregressive representations of the two time series, $x_{k,j}\left[n\right]$ and $x_{k,i}\left[n\right]$, representing the activity of the two ROIs:

(8) $$\sum_{m=0}^{p}A_{k}\left[m\right]\left[\begin{array}{c} x_{k,i}\left[n-m\right]\\ x_{k,j}\left[n-m\right] \end{array}\right]=\left[\begin{array}{c} \varepsilon_{k,i}\left[n\right]\\ \varepsilon_{k,j}\left[n\right] \end{array}\right]$$

Equation (8) derives from Equation (2) and the analog one expressing the bivariate model of $x_{k,i}\left[n\right]$; $A_{k}\left[m\right]$ are 2 × 2 coefficient matrices (identity matrix at time lag 0). After Fourier transforming Equation (8), we can manipulate it to obtain

(9) $$\left[\begin{array}{c} X_{k,i}\left(f\right)\\ X_{k,j}\left(f\right) \end{array}\right]=\left[\begin{array}{cc} H_{k,ii}\left(f\right) & H_{k,ij}\left(f\right)\\ H_{k,ji}\left(f\right) & H_{k,jj}\left(f\right) \end{array}\right]\left[\begin{array}{c} E_{k,i}\left(f\right)\\ E_{k,j}\left(f\right) \end{array}\right]=H_{k}\left(f\right)\left[\begin{array}{c} E_{k,i}\left(f\right)\\ E_{k,j}\left(f\right) \end{array}\right]$$

$H_{k}\left(f\right)=A_{k}^{-1}\left(f\right)$ is the transfer function matrix. By right multiplying each side of Equation (9) by its conjugate transpose (*), the cross-spectral density matrix $S_{k}\left(f\right)$ for signals $x_{k,j}\left[n\right]$ and $x_{k,i}\left[n\right]$, can be expressed as

(10) $$S_{k}\left(f\right)=H_{k}\left(f\right)\Sigma_{k}H_{k}{\left(f\right)}^{*}$$

where $\Sigma_{k}=\left[\begin{array}{cc} \sigma_{k,ii} & \sigma_{k,ij}\\ \sigma_{k,ij} & \sigma_{k,jj} \end{array}\right]$ is the covariance matrix of the prediction errors (white noise processes) in Equation (8). The spectral Granger causality from $x_{k,i}\left[n\right]$ to $x_{k,j}\left[n\right]$ is computed as follows (for further mathematical details see [44]):

(11) $$sGC_{k,ROI_{i}\to ROI_{j}}\left(f\right)=\ln\frac{P_{jj}\left(f\right)}{P_{jj}\left(f\right)-\left(\sigma_{ii}-\frac{\sigma_{ij}}{\sigma_{jj}}\right){\left|H_{ji}\left(f\right)\right|}^{2}}=\ln\frac{P_{jj}\left(f\right)}{P_{jj}\left(f\right)-{\;\tilde{\sigma }}_{ii}{\left|H_{ji}\left(f\right)\right|}^{2}}$$

The numerator expresses the total power spectrum of $x_{k,j}\left[n\right]$ at frequency f, whereas the denominator is the difference between the total power spectrum and the “causal” power exerted by signal $x_{k,i}\left[n\right]$ on signal $x_{k,j}\left[n\right]$ at the same frequency. Accordingly, the quantity $sGC_{k,ROI_{i}\to ROI_{j}}$ at a given frequency *f* is zero when the causal power of $x_{k,i}$ onto $x_{k,j}$ at *f* is zero and increases (>0) as the causal power increases. The spectral Granger causality from $x_{k,j}$ to $x_{k,i}$, $sGC_{k,ROI_{j}\to ROI_{i}}$, is obtained from Equation (11) by exchanging the subscripts *j* and *i*.

It is worth noting that the spectral Granger causality provides a value for each frequency. To summarize the results, we then computed a single value of connectivity from region $ROI_{i}$ to $ROI_{j}$ by evaluating the average value within the overall frequency band: 4–45 Hz. This value, named $sGC_{k,ROI_{i}\to ROI_{j}}^{tot}$, was used in subsequent analysis.

Starting from the Granger causality computed in the total frequency band, we calculated the same indices defined above (i.e., ${\tilde{GC}}_{k,ROI_{i}}^{OUT}$ (k=1,…,20) and ${\tilde{GC}}_{k,ROI_{i}}^{IN}$ (k=1,…,20)) using the same normalization procedure as before for $sGC_{k,ROI_{i}\to ROI_{j}}^{tot}$, but only with reference to the ROIs that were previously identified as “AQ-modulated source ROIs” (or “AQ-modulated sink ROIs”). Finally, for those ROIs only, we evaluated the statistical significance of the difference between the two groups using the unpaired *t*-test.

Moreover, in order to identify the contributions of the different frequency bands to Granger causality, we defined a connectivity index between regions $ROI_{i}$ and $ROI_{j}$ in each band as follows:

(12) $$sGC_{k,ROI_{i}\to ROI_{j}}^{\theta }\frac{\Delta f_{\theta }}{\Delta f_{tot}}\;\mathrm{connectivity}\;\mathrm{index}\;\mathrm{for}\;\mathrm{the}\;\mathrm{theta}\;\mathrm{band}$$

(13) $$sGC_{k,ROI_{i}\to ROI_{j}}^{\alpha }\frac{\Delta f_{\alpha }}{\Delta f_{tot}}\;\mathrm{connectivity}\;\mathrm{index}\;\mathrm{for}\;\mathrm{the}\;\mathrm{alpha}\;\mathrm{band}$$

(14) $$sGC_{k,ROI_{i}\to ROI_{j}}^{\beta }\frac{\Delta f_{\beta }}{\Delta f_{tot}}\;\mathrm{connectivity}\;\mathrm{index}\;\mathrm{for}\;\mathrm{the}\;\mathrm{beta}\;\mathrm{band}$$

(15) $$sGC_{k,ROI_{i}\to ROI_{j}}^{\gamma }\frac{\Delta f_{\gamma }}{\Delta f_{tot}}\;\mathrm{connectivity}\;\mathrm{index}\;\mathrm{for}\;\mathrm{the}\;\mathrm{gamma}\;\mathrm{band}$$

where $\Delta f_{\theta }$, $\Delta f_{\alpha }$, $\Delta f_{\beta }$, and $\Delta f_{\gamma }$ are the bandwidths of the different rhythms, and $sGC_{k,ROI_{i}\to ROI_{j}}$ with superscripts θ, α, β, and γ indicates the average values of $sGC_{k,ROI_{i}\to ROI_{j}}$ computed in the respective band. In particular, we used a theta band of 4–8 Hz, an alpha band of 8–14 Hz, a beta band of 14–30 Hz, a gamma band of 30–45 Hz, and a total band of 4–45 Hz. It is noticeable that thanks to Equations (12)–(15), the sum of the connectivities in the different bands provides the total connectivity value, i.e., we have

$$sGC_{k,ROI_{i}\to ROI_{j}}^{tot}=sGC_{k,ROI_{i}\to ROI_{j}}^{\theta }\frac{\Delta f_{\theta }}{\Delta f_{tot}}+sGC_{k,ROI_{i}\to ROI_{j}}^{\alpha }\frac{\Delta f_{\alpha }}{\Delta f_{tot}}+sGC_{k,ROI_{i}\to ROI_{j}}^{\beta }\frac{\Delta f_{\beta }}{\Delta f_{tot}}+sGC_{k,ROI_{i}\to ROI_{j}}^{\gamma }\frac{\Delta f_{\gamma }}{\Delta f_{tot}}$$

*Grahical Analysis*—Finally, we selected only the regions that were defined as AQ-modulated source ROIs and AQ-modulated sink ROIs according to the temporal Granger causality, but that also resulted in being significantly different according to the further analysis performed in the frequency domain. Then, we computed the average value, over Class1 and over Class2, of each outgoing connection (still normalized, i.e., as in Equation (5)) from the AQ-modulated source ROIs. The difference between the two classes was computed and graphed to assess how the output connectivity from the AQ-modulated source ROIs differed between the two groups. This same computation was replicated for each connection entering the AQ-modulated sink ROIs in order to assess how the input connectivity targeting these ROIs differed between the two groups of participants.

## 3. Results

As described in the Methods section, we first normalized the connectivity matrix so that the sum of all connections among the 68 brain regions was as high as 100 in each participant (Equations (4) and (5)). Then we analyzed where the connections were stronger or weaker (in the normalized scale) as a function of AQ. In particular, for each k−th subject, we focused attention on the output sum (${\tilde{GC}}_{k,ROI_{i}}^{OUT}$), i.e., the sum of the connections leaving each i−th ROI in the network, and the input sum (${\tilde{GC}}_{k,ROI_{i}}^{IN}$), i.e., the sum of the connections entering each i−th ROI. Of course, we had 68 values for both the output sum and the input sum (one value for each ROI) per participant. We assessed whether these quantities exhibited a dependence on the AQ score by computing the statistical difference between the two classes, and the regression coefficient between these quantities and the AQ score in the 20 participants and focusing only on the ROIs that exhibited a correlation coefficient higher than 0.4, a significant *p* value for testing the hypothesis of no correlation, and a significant difference in quantity ${\tilde{GC}}_{k,ROI_{i}}^{OUT}$ (or ${\tilde{GC}}_{k,ROI_{i}}^{IN}$) between the two classes.

**Figure 1:**
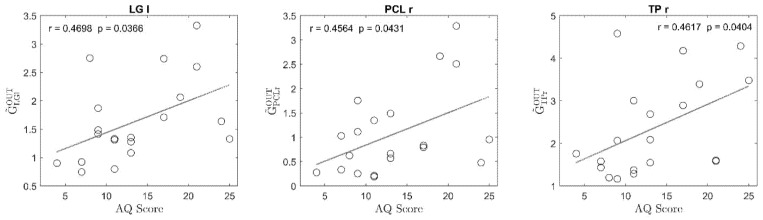
Plot of the regression lines between the sum of the normalized connections exiting from a given region (output sum ${\tilde{GC}}_{ROI_{i}}^{OUT}$) and the Autism Spectrum Quotient (AQ) score, obtained using data from the 20 participants. Only plots in the three regions that exhibit a correlation coefficient higher than 0.4, with a significant *p* value for the correlation and significant difference between the two classes, are shown. For each plot, the regression coefficient and the *p* value for testing the hypothesis of no-correlation are shown in the label. It is worth noting that to obtain these plots, the connection matrix for each participant was normalized so that the total sum of connection strength was as high as 100 (see Equations (4)–(6)); hence, for example, a value of 3 on the *y*-axis indicates that 3% of the total connections in the network are exiting from that particular ROI. Hence, the values in these plots reflects whether the connections leaving the ROI are stronger (high value) or weaker (low value) compared to the total sum of the connections in the same subject.

The results concerning the quantity of the output sum are illustrated in Figure 1. In this case, three ROIs were AQ-modulated as sources of casual influence, since they satisfied all three requisites: they were the LG left, PCL right, and TP right (see the glossary for the meaning of the names). From the plots we can conclude that the overall connectivity outflowing from these ROIs was stronger in participants with higher autistic traits; moreover, in each of these regions the *p* value for testing the hypothesis of no correlation (Bonferroni uncorrected) was lower than 0.05.

**Figure 2:**
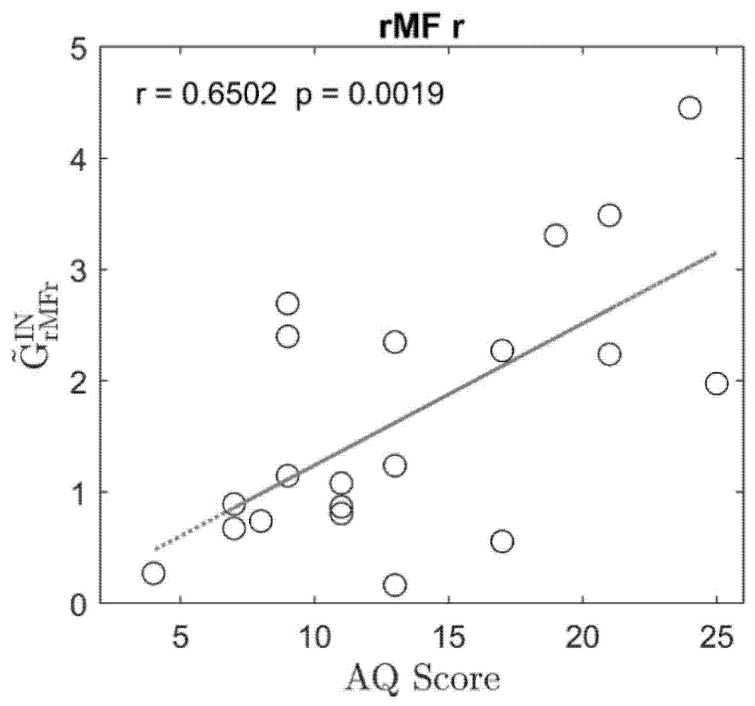
Plot of the regression line between the sum of the normalized connections entering a given region (input sum ${\tilde{GC}}_{ROI_{i}}^{IN}$) and the Autism Spectrum Quotient (AQ) score, obtained using data from 20 participants. Only plots in the region that exhibit a correlation coefficient higher than 0.4, with a significant *p* value for the correlation and a significant difference between the two classes, are shown. In the plot, the regression coefficient and the *p* value for testing the hypothesis of no-correlation are shown in the label. It is worth noting that to obtain this plot, the connection matrix for each participant was normalized so that the total sum of connection strength was as high as 100 (see Equations (4), (5) and (7)); hence, for example, a value of 3 on the *y*-axis indicates that 3% of the total connections in the network are entering that particular ROI. Hence, the values in the plot summarize whether the connections entering the node are stronger (high value) or weaker (low value) compared to the total sum of the connections in the same participant.

The results concerning the quantity of the input sum are illustrated in Figure 2. In this case, just one region was AQ-modulated as a sink of causal influences, exhibiting a correlation higher than 0.4, a significant *p* value for testing the hypothesis of no correlation, and a statistical significance between the two classes; this was the rMF right, for which the overall inflow connectivity was higher in participants with higher autistic traits, with an uncorrected *p* value of 0.0019.

**Figure 3:**
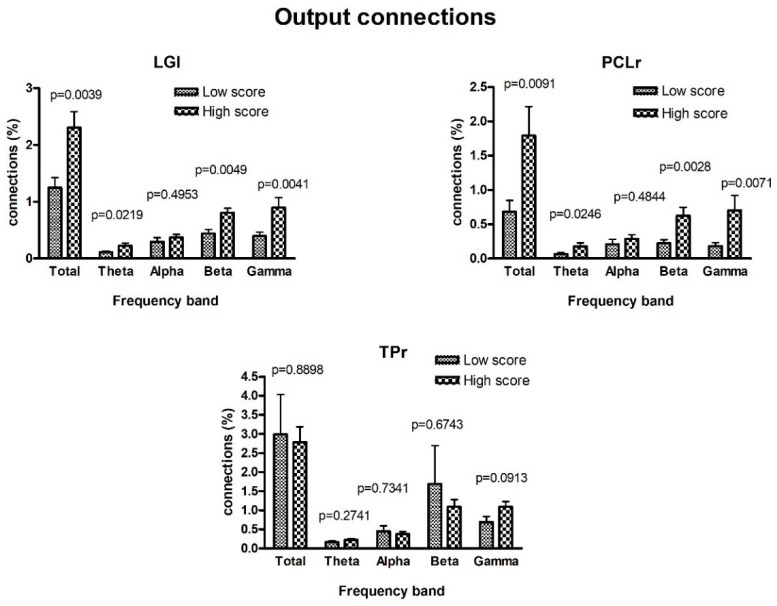
Comparison between the normalized connections exiting from a given region $ROI_{i}$ in the two classes of subjects with lower and higher AQ scores, obtained using the spectral Granger connectivity. The comparison is performed using the overall connectivity computed in the entire frequency range ($sGC_{k,ROI_{i}\to ROI_{j}}^{tot}$) and the connectivity in the individual bands, with reference to the three regions defined as AQ-modulated source ROIs in Figure 1. Note that differences in TPr are not significant using spectral Granger causality instead of the temporal Granger causality. Moreover, differences in LGl and PCLr are highly significant in the beta and gamma bands, but not in the alpha band.

**Figure 4:**
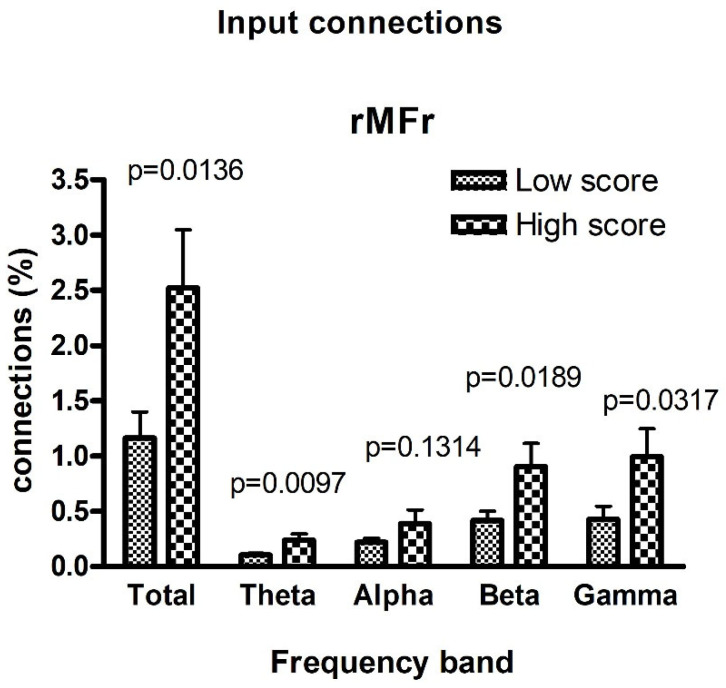
Comparison between the normalized connections entering a region $ROI_{i}$ in the two classes of subjects with lower and higher AQ scores, obtained using the spectral Granger connectivity. The comparison is performed using the overall connectivity computed in the entire frequency range ($sGC_{k,ROI_{j}\to ROI_{i}}^{tot}$) and the connectivity in the individual bands, with reference to the unique region defined as an AQ-modulated sink ROI in Figure 2. Note that differences in the region rMFr are significant in the theta, beta, and gamma bands, but not in the alpha band.

Figure 3 and Figure 4 show a comparison between the frequency Granger connectivity values obtained in the two groups (mean values and standard deviation) for what concerns the normalized connections exiting from a given region (Figure 3) or entering a given region (Figure 4). The comparison is limited to the regions previously identified in Figure 1 (AQ-modulated source ROIs) and Figure 2 (AQ-modulated sink ROIs). Both the total connectivity in the overall band of 4–45 Hz and the connectivity values within the single bands are shown, together with the *p* value (uncorrected) of the statistical difference.

Significant statistical differences are evident for what concerns the regions PCLr and LGl in Figure 3. Moreover, these differences are significant in the theta band and highly significant in the beta and gamma bands, but are not significant in the alpha band. Conversely, no significant difference was observed for the TPr region, at odds with what has been previously obtained using the temporal Granger causality (Figure 1). Hence, the latter region was excluded from the subsequent graphic plot.

In Figure 4, the unique region (rMFr) identified as an AQ-modulated sink ROI exhibited highly significant statistical differences between the two groups in the theta, beta, and gamma bands, but not in the alpha band.

In conclusion, after the analysis with the frequency Granger connectivity, only two regions (PCLr and LGl) remained as AQ-modulated source ROIs, and a single region (rMFr) as an AQ-modulated sink ROI.

**Figure 5:**
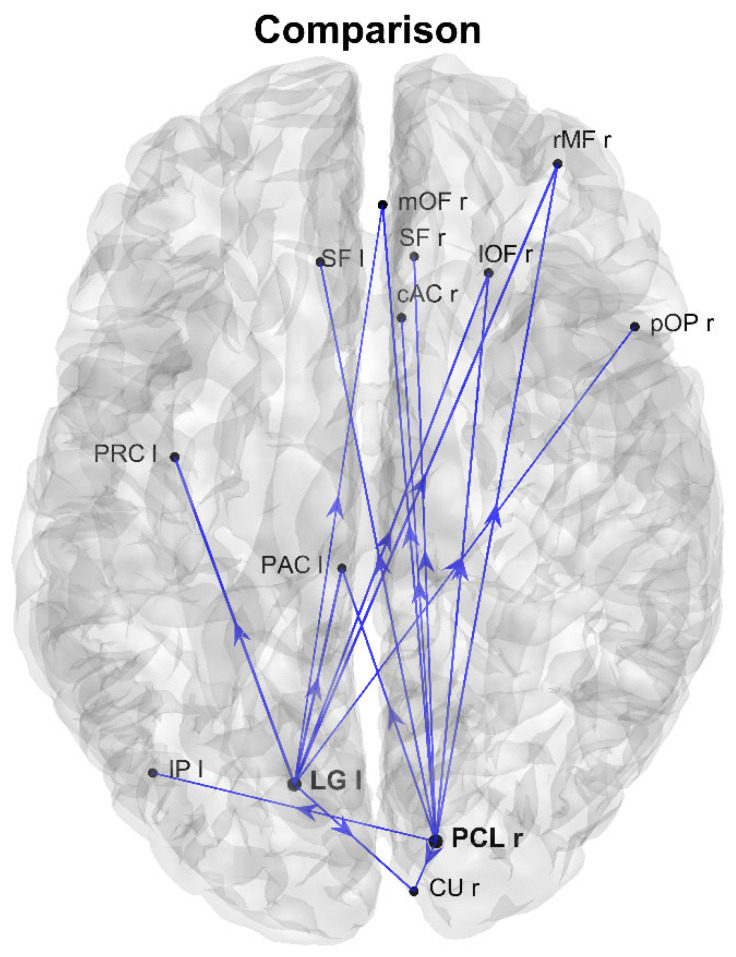
Network showing the differences between the normalized connections in the two groups, with reference to the connections that exit from the AQ-modulated source ROIs identified in Figure 1, with the exclusion of TPr, not significant in Figure 3. Only differences with an absolute value higher than 0.025 are shown. Blue lines denote connections higher in the group with higher autistic scores (AQ score ≥ 17). The presence of two nodes (LG left and PCL right) with stronger output connectivity is evident in the group with higher autistic traits.

Figure 5 shows the differences in the connections that exited from the two AQ-modulated source regions previously identified from Figure 1 and Figure 3 (we eliminated the TPr region, which was not significant according to the Granger analysis in the frequency domain). Blue lines indicate a stronger connection in the group with higher autistic traits (only the connections with an absolute value difference between the two groups higher than 0.025 are plotted). As is clear from this figure, one can observe that the connections exiting from these two regions were much stronger in the group with higher autistic traits; moreover, most of the connections from LG left and PCL right (Out degrees as high as 7 and 9, respectively) were directed from the occipital toward the higher fronto-parietal regions.

**Figure 6:**
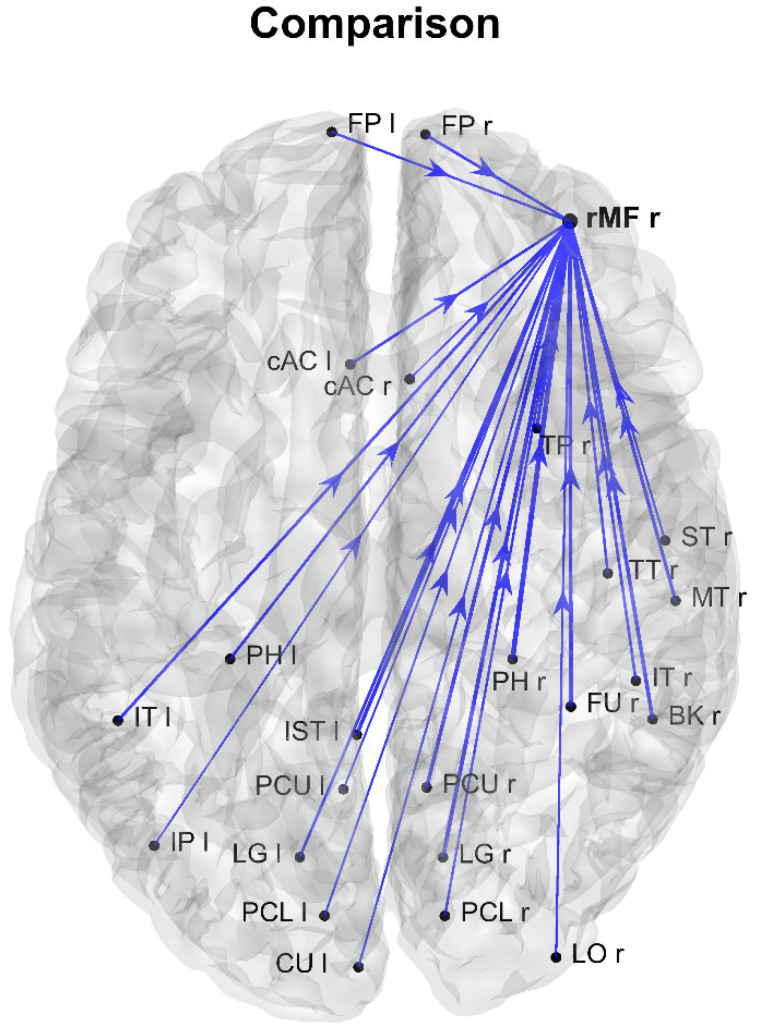
Network showing the differences between the normalized connections in the two groups, with reference to the connections that enter the unique AQ-modulated sink ROI identified in Figure 2 (and confirmed by Figure 4). Only differences with an absolute value higher than 0.025 are shown. Blue lines denote connections higher in the group with higher autistic scores (AQ score ≥ 17). It is evident that the ROI rMF right is characterized by stronger input connections in the group with higher autistic scores relative to the group with lower autistic scores.

Figure 6 shows the differences in the connections that entered the unique AQ-modulated sink ROI previously identified in Figure 2. In this case, too, only differences higher than 0.025 (in absolute value) are shown, with blue lines denoting stronger connections in the group with higher autistic traits. In this group, the rMF right region exhibited a large number of stronger entering connections (In degree 24), and most of those connections originated from lower occipital-parietal regions.

## 4. Discussion

In the present study we demonstrated the presence of a differential connectivity pattern according to autistic traits in a non-clinical population. To the best of our knowledge, this is the first study that has exploited Granger causality analysis to investigate the brain organization connected to autistic features in a resting-state electroencephalographic study. Although there is abundant evidence of abnormal brain connectivity in clinical forms of autism, there are limited studies investigating whether such abnormalities are also traceable in the general population with high autistic traits [30].

In literature, ASD has been framed as either a disconnection or over-connection disorder [45]. Recent experimental evidence has shown that both hypotheses may be appropriate if weighted according to the directionality of the considered connectivity. Specifically, in ASD there would be the presence of increased functional connectivity in the feed-forward direction and hypoconnectivity in the feedback direction [25]. The employment of a directional functional connectivity method like Granger causality has allowed us to investigate the existence of a direction-dependent connectivity pattern that mimics that observed in ASD, even in the general population, depending on the autistic trait.

An important aspect of our analysis, compared with previous connectivity studies, is that we investigated the *normalized* connectivity network, i.e., the sum of the connectivity values was equal to 100 in each subject. This choice allowed us to understand whether a given subject made greater or lesser use of connections in a particular direction compared to other parts of his/her brain connectivity network, and to summarize which hubs are particularly relevant for information processing (considered either as a source to generate information to be transmitted toward other regions, or as sink to collect information from different regions). The basic idea is that a connectivity strength is not important per se, but compared with the strength of other connections within the same network, to decide where information is principally created, transmitted, and manipulated within a complex and strongly interconnected processing system.

The conducted analysis shows that connectivity along the fronto-posterior axis appears to be sensitive to the magnitude of individual autistic traits. Indeed, high-AQ individuals are characterized by a prevalence of connections that originate from posterior brain regions ascending the cortical hierarchy in a feed-forward flow.

In particular, in the high-AQ group, there was a greater number of connections entering rMF right, deriving mainly from the occipito-parietal areas. rMF encapsulates several frontal Brodmann regions (including BA46; [46]) critical for higher-order executive functions, including working memory, attention, and decision-making [47]. Thus, as the autistic trait increases, the areas involved in executive control are overloaded with information deriving from the lower regions involved in sensory processing. This preponderance of signals coming from the posterior region of the brain is further supported by the finding that the ROIs in which the outgoing connections are greater in the high- compared to the low-AQ score are mainly of occipital origin (i.e., LG left and PCL right). Crucially, most of the connections exiting from these regions are directed toward fronto-parietal hubs. This pattern of results resembled that observed in clinical forms of autism. In a resting-state functional MRI study, Keehn et al. [48] showed that a subgroup of adolescents with ASD was characterized by occipito-frontal overconnectivity, which was also associated with autism symptom severity. Interestingly, the occipito-frontal seeds associated with over-connectivity in their study (i.e., pericalcarine cortex and middle frontal gyri) were similar to those shown in our study. Moreover, individuals along the autistic spectrum performed better on tasks relying on bottom-up processing and poorly on tasks where top-down coordination is required, and this behavioral pattern was paired with a higher activity in the occipital and posterior parietal cortex and lower activity in the frontal cortex [49,50].

We further investigated at the spectral level which frequency bands support this enhanced forward signaling of neural information. The conducted Granger spectral analysis showed that for all three AQ-modulated ROIs (i.e., rMFr, LGl, and PCLr), the increased connectivity in high-AQ individuals was bound to the theta, beta, and gamma bands, but not to the alpha band. Overall, these results resemble the U-shaped curve that marks power anomalies in the ASD population (i.e., greater activity in lower and higher frequencies) [29]. It is of considerable interest to point out that the higher frequencies explain most of the variance in the total connectivity, whereas the alpha band explains relatively less, despite being the dominant rhythm at rest (Figure 3 and Figure 4). This may result from the prevailing directionality of the connections embedded in this analysis (i.e., low to high). Indeed, higher frequencies like gamma have been linked specifically to feed-forward connectivity [51]. Thus, this hypersignaling along the gamma band further supports the hypothesis of a tendency towards ascending processing on the autistic spectrum. Moreover, this could also explain why no significant results emerged in the alpha band, as it is more involved in feedback processing [27,52], although when looking at posterior alpha signals, we found that participants with high scores at AQ manifested a significantly lower alpha amplitude relative to low-AQ participants (see Supplementary Materials). On the other hand, we also found higher connectivity in the theta band and especially for signals entering frontal areas. This also seems to be in line with data reported in the literature concerning ASD with typical enhancement in the frontal theta band, possibly signaling an exaggerated cognitive control over poor predictive resources [28]. Another cognitive feature observed in ASD that is related to enhanced bottom-up processing is the bias towards local information processing [53]. In visual search tasks in which the target differs from distractors by small details, ASD individuals show above-average performance, relying on an abnormally intensified perception of stimulus particularities [54,55]. This greater reliance on details was also observed in neurotypical individuals with an above-average number of autistic traits [10]. Crucially, beta oscillations have been linked to local feature processing [56,57] and beta hyperconnectivity to detail-oriented processing [58], and greater occipitofrontal coherence in beta bands was positively correlated with the attention to details score [59]. Therefore, future studies might assess whether this beta hyper-synchronization recorded at rest is associated with abnormalities in local vs. global information processing as a function of AQ score.

Another relevant aspect concerns the generalizability of our findings to the clinical sample of ASD. Indeed, although there is an imbalance in the clinical population between males and females that is not strictly maintained in the proposed study, there are many similar studies employing a predominantly female sample (e.g., [10,60]) where a higher level of autistic traits correlated with the presence of a behavioral and neural pattern that closely resembles that found in clinical forms of autism. Therefore, this aspect of the study should not interfere with the potential generalization of the results. Furthermore, we provide evidence (see Supplementary Material) that our dataset resembles the resting EEG characteristics typical of the ASD population (e.g., [61,62]), showing that the differences noted in our study between high- vs. low-AQ participants are comparable to those found in other studies between ASD and controls. This demonstrates that our high-AQ sample exhibited characteristics typical of the ASD population, increasing the possibility that the connectivity peculiarities that emerged in our sub-clinical sample can be generalized to clinical variants of the spectrum as well.

Important and novel aspects of our results are that these highlighted characteristics in brain organization appear to be present even at rest and to involve some specific ROIs. This would testify to the presence of an intrinsic and stable bias within specific neural networks in the signaling of bottom-up information in people with high autistic traits.

However, it is fair to note that in this study the emergence of some specific ROIs was not based on a statistical comparison of the 68 ROIs across high- and low-AQ-score subjects, with multiple comparison correction (the very high number of comparisons strongly limits the possibility of obtaining significant results). Rather, ROI selection was based on the value of the correlation coefficient (to identify a dependence between ROI connectivity and AQ score) rather than on the corrected *p* value. The adopted procedure should be considered as a way to generate new and preliminary hypotheses (according to the obtained results), and follow-up studies using larger sample size are needed to test and validate them, not only examining resting-state conditions but possibly also task conditions. Indeed, since some behavioral patterns observed in ASD are also found in individuals with high autistic traits, it would be of great interest to investigate whether and how the pattern of altered directionality observed here in resting-state connectivity along the autism continuum also extends during task performance (e.g., during decision-making tasks), providing a neural substrate underlying the noted similarities at the behavioral level. Finally, although it would have been extremely valuable to analyze the contribution that the different subscales had towards the connectivity measures examined, this investigation would have significantly inflated the number of comparisons against our sample. Thus, our findings provide novel insights that will need to be extended in future studies by specifically investigating the contribution that each subscale has with respect to our current findings and their impact on behavior.

## 5. Conclusions

To sum up, the obtained findings suggest the presence of a hyper-connectivity pattern along the posterior-anterior gradient as individual autistic traits increase. Indeed, in individuals with a higher AQ score, the anterior brain areas are overwhelmed by information coming from downstream areas. This indicates that peculiarities in brain organization are not only traceable in diagnosed autism, supporting the idea of a neurobiological continuum between autistic traits and clinical autism spectrum conditions.

## Figures and Tables

**Table 1:** The 68 ROIs defined by the Desikan–Killiany Atlas provided in the Brainstorm software and the corresponding abbreviation.

ROI	Abbreviation	ROI	Abbreviation
Banks of Sup. Temp. Sulcus	BK	Parahippocampal	PH
Caudal Anterior Cingulate	cAC	Pars Opercularis	pOP
Caudal Middle Frontal	cMF	Pars Orbitalis	pOR
Cuneus	CU	Pars Triangularis	pTR
Entorhinal	EN	Pericalcarine	PCL
Frontal Pole	FP	Postcentral	POC
Fusiform	FU	Posterior Cingulate	PCG
Inferior Parietal	IP	Precentral	PRC
Inferior Temporal	IT	Precuneus	PCU
Insula	IN	Rostral Anterior Cingulate	rAC
Isthmus Cingulate	IST	Rostral Middle Frontal	rMF
Lateral Occipital	LO	Superior Frontal	SF
Lateral Orbitofrontal	IOF	Superior Parietal	SP
Lingual	LG	Superior Temporal	ST
Medial Orbitofrontal	mOF	Supramarginal	SMG
Middle Temporal	MT	Temporal Pole	TP
Paracentral	PAC	Transverse Temporal	TT

## Data Availability

The data presented in this study are available on request from the corresponding authors.

## Supplementary Materials

The following are available online at https://www.mdpi.com/article/10.3390/brainsci11111443/s1, Figure S1: Mean log-power spectrum in the group of individuals with high autistic traits (*n* = 7) and low autistic traits (*n* = 13), Figure S2: Relative Alpha Power (Oz), *p* = 0.048.
