# Supplementary material for: The Directionality of Fronto-Posterior Brain Connectivity Is Associated with the Degree of Individual Autistic Traits
Source: Brain Sci. 2021 Oct 29;11(11):1443. doi: 10.3390/brainsci11111443 (PMC8615575; doi:10.3390/brainsci11111443)
Supplement: Supplementary file 1 [file brainsci-11-01443-s001.zip › brainsci-1311585-supplementary.pdf]

Extracting Power Spectral Densities (PSD)

We calculated power spectral densities (PSD) using “pwelch” function in MATLAB at Oz electrodes. For PSD estimation, we chose a 2-s Hann window with a 50% overlap between segments. Then, the resulting power spectrum were log transformed (**Fig. S1**).

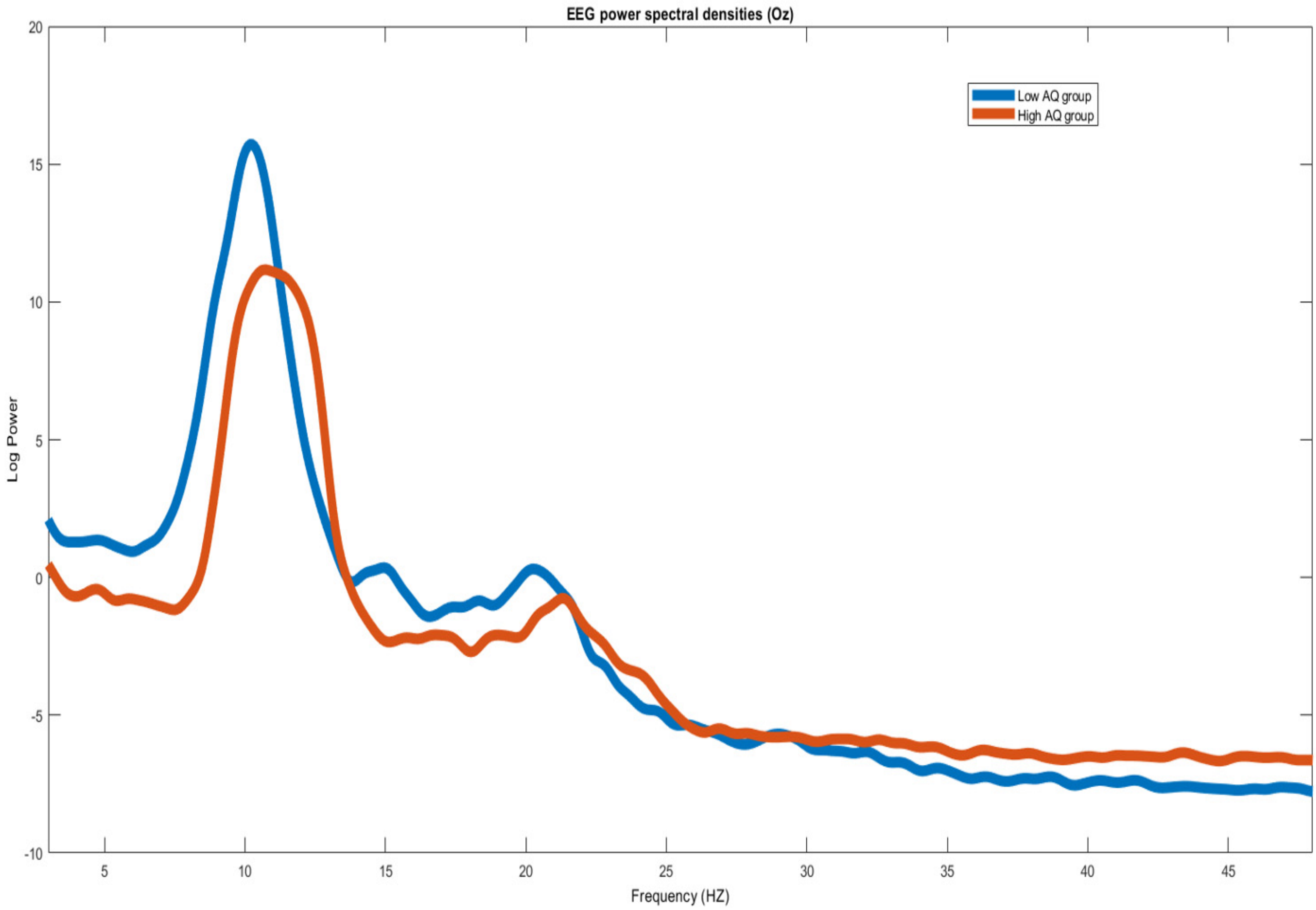

Figure S1. Mean log-power spectrum in the group of individuals with high autistic traits (n = 7) and low autistic traits (n = 13).

Extracting Relative Alpha power

PSDs were transformed into relative powers so that spectral activities from all individuals were set to an equal footing for comparison. The relative alpha powers were calculated dividing the sum of the power between 8 and 12 Hz with the sum of the power between 1 and 50 Hz. Next, we compared whether the level of occipital alpha power shows differentiation according to autistic traits. Thus, we assess the presence of significant differences in alpha power in the Oz electrode between the high autistic traits group (n = 7) and the low autistic traits group (n = 13). The conducted t-test shows the presence of a significant difference between the two groups ($t(18) = 2.12$; $p = .048$). Specifically, the low-AQ group show increased occipital alpha power relative to high-AQ group (**Fig. S2**). Crucially, this differentiation resembles the difference observed between the control group and ASD [1,2].

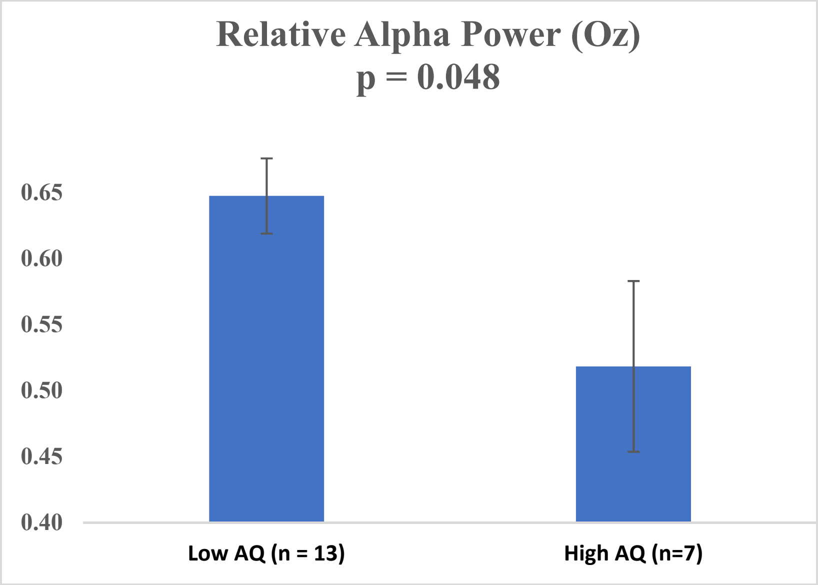

Figure S2

Reference

1. Bellato, A.; Arora, I.; Kochhar, P.; Hollis, C.; Groom, M.J. Atypical Electrophysiological Indices of Eyes-Open and Eyes-Closed Resting-State in Children and Adolescents with ADHD and Autism. *Brain Sciences* **2020**, *10*, 272, doi:10.3390/brainsci10050272.
2. Mash, L.E.; Keehn, B.; Linke, A.C.; Liu, T.T.; Helm, J.L.; Haist, F.; Townsend, J.; Müller, R.-A. Atypical Relationships Between Spontaneous EEG and FMRI Activity in Autism. *Brain Connectivity* **2020**, *10*, 18–28, doi:10.1089/brain.2019.0693.
